# Five foundational tools for managing metadata from the USDA Long‐Term Agroecosystem Research (LTAR) Network

**DOI:** 10.1002/jeq2.70027

**Published:** 2025-04-25

**Authors:** Nicole E. Kaplan, Gerardo Armendariz, Shefali Azad, Bryan R. Carlson, William A. White, Lori J. Abendroth, Alisa W. Coffin, Vanessa S. Gordon, Jude E. Maul, William Osterholz, Jonathan Sears

**Affiliations:** ^1^ USDA‐ARS Rangeland Resources and Systems Research Unit Fort Collins Colorado USA; ^2^ USDA‐ARS Southwest Watershed Research Center Tucson Arizona USA; ^3^ Archbold Biological Station Venus Florida USA; ^4^ USDA‐ARS Northwest Sustainable Agroecosystems Research Unit Pullman Washington USA; ^5^ USDA‐ARS Hydrology and Remote Sensing Laboratory Beltsville Maryland USA; ^6^ USDA‐ARS Cropping Systems and Water Quality Research Unit Columbia Missouri USA; ^7^ USDA‐ARS Southeast Watershed Research Laboratory Tifton Georgia USA; ^8^ USDA‐ARS National Agricultural Library, Information Products Division Beltsville Maryland USA; ^9^ USDA‐ARS Sustainable Agricultural Systems Laboratory Beltsville Maryland USA; ^10^ USDA‐ARS Soil Drainage Research Unit Columbus Ohio USA; ^11^ USDA‐ARS, National Agricultural Library, Knowledge Services Division Beltsville Maryland USA

## Abstract

The United States Department of Agriculture Long‐Term Agroecosystem Research (LTAR) Network comprises 19 sites and has collectively produced nearly one petabyte of data. Data include time‐series measurements, remotely sensed imagery, and high‐throughput environmental data from field and laboratory instrumentation. Currently, network‐level analyses leverage multi‐decadal data from historical, as well as ongoing, and coordinated data collection from several network sites. Though this multifaceted data facilitates analyses on cross‐site, regional, and national levels, its analytical power is constrained by the locally organized and siloed management and storage practices in place. A network information management system is crucial for robust meta‐analyses and syntheses exploring the agricultural management impacts on agroecosystems production, structure, and function across the various LTAR sites. Foundational tools described herein provide the framework for an LTAR network information system that will empower users to find, harmonize, map, and share data across all network locations. Standard metadata have been created and implemented for (1) inventorying datasets managed by each site, (2) creating controlled vocabularies for measurements to facilitate cross‐site comparisons and analyses, (3) geolocating data collection, site, and experimental boundaries, (4) publishing protocols to describe how data were generated, (5) reporting the quantitative research impact of published literature, and (6) using dashboards to visualize the data collection. These efforts serve as a pivot point around which collective work at cross‐site, regional, and national levels can occur. Harmonized data and metadata provide a robust foundation for the development of network information management and synergistic data science solutions.

## Core Ideas

- The long‐term agroecosystem (LTAR) Network has a rich legacy of data and continues to collect data for use within the network and beyond.
- Re‐use of data across research sites requires well‐described and organized metadata with standard content and structure.
- Tools for finding, harmonizing, and geolocating data rely on implementation of metadata and data standards.
- Development of standards and tools requires consensus among experts and partners.
- Standards and tools are foundational for data management to support LTAR science.

**Abbreviations**
ADC: Ag Data Commons
DOI: digital object identifier
ESRI: Environmental Systems Research Institute
ISO: international organization for standardization
LTAR: Long‐Term Agroecosystem Research
NAL: National Agricultural Library
PDI: Partnerships for Data Innovations

## 1 INTRODUCTION

**FIGURE 1:**
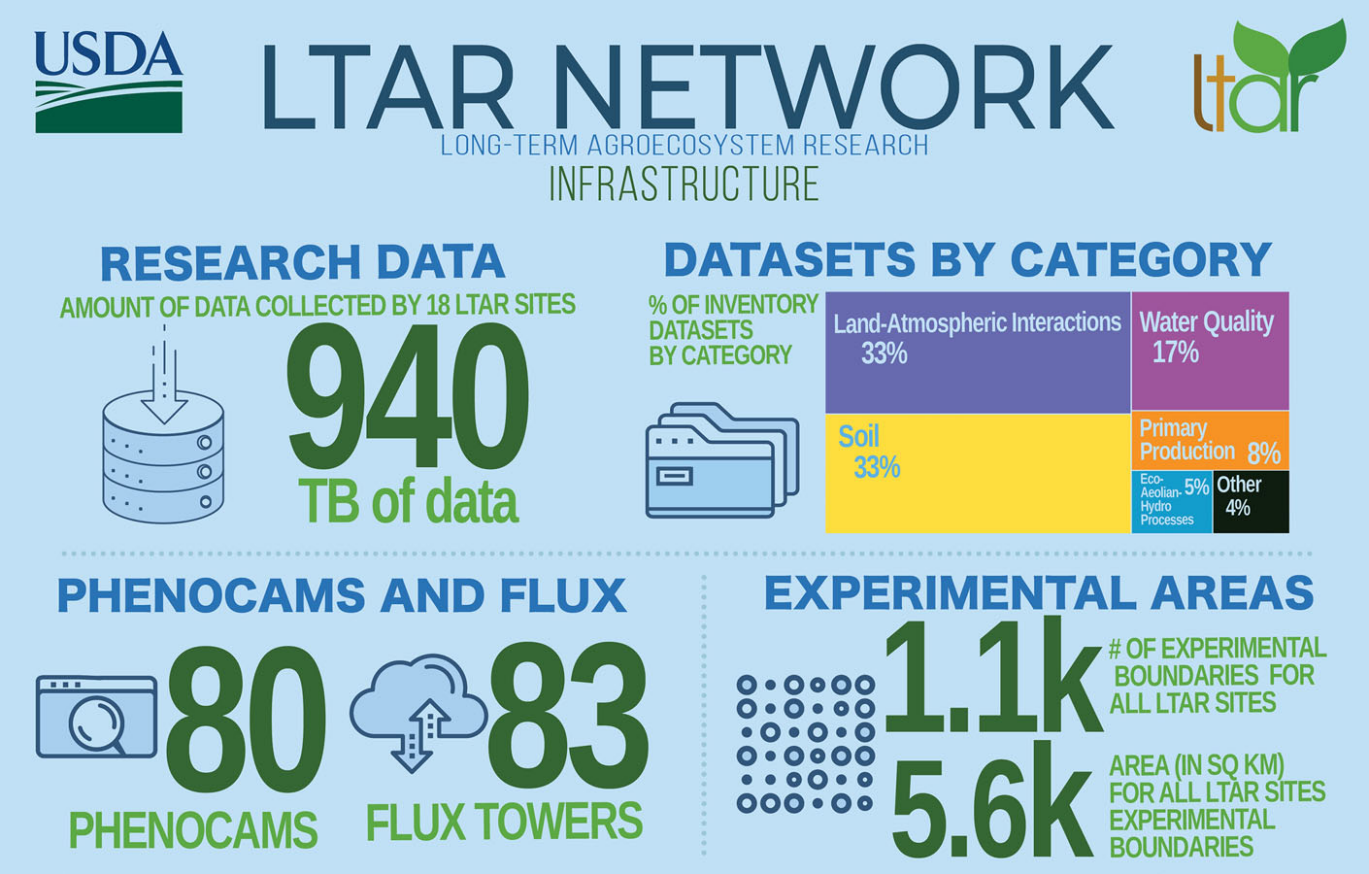
The Long‐Term Agroecosystem Research (LTAR) Network comprises 19 sites in different agroecosystems and collectively has produced nearly one petabyte of data. These data include time‐series measurements, remotely sensed imagery, and high‐throughput environmental data from field and laboratory instrumentation.

The United States Department of Agriculture (USDA) Long‐Term Agroecosystem Research (LTAR) Network (https://ltar.ars.usda.gov/) comprises 19 sites with data collections spanning historical time‐series measurements representing the research legacy at each site to current and ongoing data collected in support of Network science. The LTAR Network organizes research in accordance with its mission objectives and the strategic plans of the USDA Agricultural Research Service (ARS), the Network's host agency, resulting in substantial long‐term data collections. Current data holdings across LTAR are estimated to be approaching one petabyte and continue to increase (Figure 1). Ideally, these data holdings would be made accessible to both the network and external users from within a centralized data portal or network information system (Baker et al., 2000; Gries et al., 2023; Thibault et al., 2023; Waide et al., 2017). However, individual sites within the LTAR Network have predominantly managed their datasets in local, siloed systems, without common standards for organizing and describing them. This practice has limited harmonization of data and metadata across LTAR and created network‐wide data access challenges. Additionally, the extra‐network international agricultural and natural resources research community has a growing interest in locating and integrating long‐term data to develop evidence‐based strategies designed to ensure the coexistence of environmental quality and the production of food, fuel, and fiber (Khanna et al., 2018).

The details of data collected from the 19 LTAR Network sites are essential because each site location has a unique working landscape. Coordination of data collection and analyses across LTAR is predicated by a common conceptual framework, the “Common Experiment,” that organizes research efforts by assessing both prevailing and alternative management practices unique to each agroecosystem at a given experimental site (Kleinman et al., 2018; Liebig et al., 2024). The Common Experiment is designed to facilitate evaluation across the two treatments (i.e., prevailing and alternative), despite the agricultural management practice treatments being place‐based, highly contextual, and influenced by local socioecological conditions (Robertson et al., 2008; Waller & Flader, 2010). As the Common Experiment has been operationalized, scientists and data managers have addressed the challenges of establishing comparable metrics across the differing working landscapes and measurement approaches.

### 1.1 Metadata and standards

Metadata are required to ensure data are “Findable, Accessible, Interoperable and Reusable” (FAIR) (Wilkinson et al., 2016). Metadata elements assigned to research datasets typically fall under one of three forms: descriptive (e.g., title, author(s), abstract/description, date range, location/geospatial, and keywords), structural (e.g., related assets/publications, parent/child/collection relationships, LTAR Network sub‐group), and administrative (e.g., funding, license, and parent project/ARS National Program). In data repositories, including the USDA National Agricultural Library's (NAL) Ag Data Commons (ADC) (agdatacommons.nal.usda.gov), typical metadata records are required and checked for accuracy and completeness in accordance with the applicable metadata schema for the repository. However, for agroecosystem research data, metadata must expand its descriptors to include definitions of variables and experimental treatments, units of measure, and collection times or sampling frequencies. Contextual details of both agricultural management practices (i.e., dates of planting, harvesting, grazing, or prescribed burning) or environment (i.e., depth of soil horizons, ecological sites, topography, field, or pasture size) are important to understanding and using the data. This additional metadata best serves a collaborative research network when organized as standard content and structure (Michener et al., 1997). For example, GeoJSON format files can be used to capture site locations, and data dictionaries define data (Buchanan et al., 2021), which can be ingested in the ADC as supplemental materials. Furthermore, metadata can be used in controlled vocabularies (Porter, 2019) and community lexicons. Standardized protocols can be curated and granted a digital object identifier (DOI) for citation within published manuscripts and data sets, as well as provide adequate details for use within scientific communities (Teytelman et al., 2016).

**Core Ideas**

- The long‐term agroecosystem (LTAR) Network has a rich legacy of data and continues to collect data for use within the network and beyond.
- Re‐use of data across research sites requires well‐described and organized metadata with standard content and structure.
- Tools for finding, harmonizing, and geolocating data rely on implementation of metadata and data standards.
- Development of standards and tools requires consensus among experts and partners.
- Standards and tools are foundational for data management to support LTAR science.

### 1.2 Tool development for inventorying, visualizing, and reporting

**FIGURE 2:**
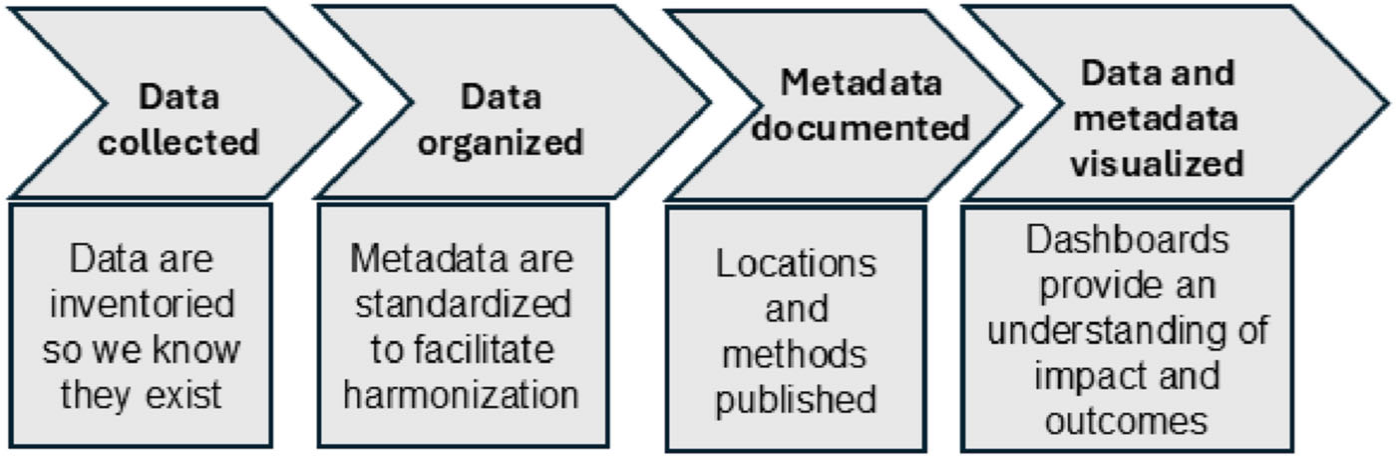
Tools are designed to meet needs in the research process that are related to essential data management steps, including data collection, organization, documentation, and publication.

In research networks, design and utilization of tools are based on community defined standards (Millerand & Baker, 2010) and provide a means to inventory, geolocate, describe, and visualize data. Tools can aid in data management for a more efficient research process at various steps, including data collection, organization, documentation, and publication (Figure 2). Public‐facing tools (e.g., dashboards) can be used for reporting and assessing impact, manipulating and visualizing data, and maximizing data relationships and comprehension. Organizing and presenting data and metadata can be challenging for interdisciplinary scientists due to the variety of methodologies, terminologies, and measurement units implemented across domains. Today, data dashboards, such as those created in Tableau, Microsoft Power BI, or Environmental Systems Research Institute (ESRI) ArcGIS Dashboards, can be embedded in websites and other virtual communications forums to produce informative reports. Data dashboards can also be included in contemporary knowledge systems, which are designed for discovery that goes beyond publications (Short et al., 2023).

### 1.3 Importance of partnerships

Data dashboards and visualizations help with communication and clarity, but data management continues to be a wicked problem for team science (Awre et al., 2015), particularly for collaborative interdisciplinary research endeavors, such as the LTAR Network. The broader organization of USDA, where LTAR is situated, can provide additional support and solutions via shared infrastructure and expertise helping to make data management tasks more tractable. The USDA includes NAL, which is one of five national libraries of the United States. It has one of the world's largest collections devoted to agriculture and its related sciences and facilitates public access to USDA‐funded research through PubAg (pubag.nal.usda.gov) and ADC (agdatacommons.nal.usda.gov). Institutional partners are essential in leveraging solutions to address data management challenges (Kaplan et al., 2021). NAL and ARS Partnerships for Data Innovations (PDI; Kharel et al., 2022) provide expertise in information and data management instruments for developing tools for LTAR.

### 1.4 Problem statement

To respond to the challenges of managing historical and current data from numerous individual sites for access and use at the LTAR Network level, four important scientific considerations need to be addressed. First, the context of the data collection is vital to its usability, demonstrating the vital need for robust metadata. Second, standards and tools are needed that facilitate the discovery, use, and re‐use of data across LTAR. Third, visualization of the data is essential to fully understand its relevance and applicability. Fourth, successful partnerships can be cultivated to fill gaps in data and metadata infrastructure and expertise for its downstream curating and publishing.

## 2 MATERIALS AND METHODS

Data managers in LTAR, with partners at NAL, collaborated to design and build tools for the discovery, harmonization, and visualization of data and metadata for the network. Scientific computing languages (i.e., R and Python) were utilized to normalize (Wickham, 2014) and analyze information and data. Various cloud‐based applications were employed to inventory, generate, and manage geospatial data, as well as create interactive dashboards. LTAR Network data resources were created through an exploratory and iterative process, gradually building elements through design, testing, evaluation, and implementation. The LTAR Network identified a set of general tools that could be applied to build network capacity and allow for creation of FAIR data. Specific requirements of these tools were defined with detailed objectives to ensure a foundation for downstream data harmonization and sharing within and outside the network. Unique reference keys were generated for components to enhance programmatic crosswalk and linkage with additional data and metadata. The supplemental information provided by integration of these tools was essential to inform network users on attributes of measurements collected, including methodologies employed and locations of data collected.

Tool development in the LTAR Network has been focused on (1) inventorying datasets managed by each site, (2) creating controlled vocabularies for measurements to promote cross‐site comparisons and analyses, (3) geolocating data collections, site, and experimental boundaries, (4) publishing data‐generation protocols, (5) reporting quantitative (e.g., productivity as number of publications) and qualitative (e.g., number of citations) research impacts of published literature, and (6) using dashboards to visualize data availability, location, and collection period.

### 2.1 Inventorying and describing data

The purpose of the LTAR data inventory is to make data and metadata from all LTAR sites discoverable. The notion of data inventory was conceived at the February 2016 LTAR Annual Science Meeting at the Archbold Biological Station in Venus, Florida, to address a gap in identifying which sites produced what data. The first version of data inventory was presented at the June 2019 LTAR Annual Science Meeting in Lincoln, Nebraska. A common data entry template was created for sites to enter the following attributes for legacy measurements: variable names and definitions, units, dataset temporal extent as start and end dates for collection, frequency of measurements taken, data contributor's contact information, storage format (e.g., comma‐delimited), storage repositories (e.g., local relational database management system or DOI in an institutional repository), data spatial extent as descriptive text of the field site, and number of instruments or collection points to understand the number of replications or statistical unit of analysis. An R script (R Core Team, 2021) was used to read each site's inventory from Google Sheets and create an appended network inventory. Because each site used unique names for their measurements, categories reflecting scientific domains within the network were used to aggregate data for display in a dashboard using ESRI ArcGIS Insights. In May 2020, the LTAR data inventory team formalized and identified a set of International Organization for Standardization (ISO) compliant terms to describe relevant attributes in an extended data dictionary. This effort used a combination of R scripts and manual manipulations to convert dates, units, and frequency of measurements to standard ISO formats. A description of the measurement was made a requirement.

To better position this data inventory as a network resource (NSB, 2005), two subsequent versions were developed. Version 2 was imported into Airtable, a cloud‐based relational database platform, which allowed for better row‐level version control. The data inventory could be linked with other data management efforts, embedded on the LTAR Network website using Airtable application programming interface, and incorporated into a dashboard using Tableau. Additionally, measurements were mapped to a new conceptual framework developed within the network, that of sustainable indicators of agricultural intensification, which would eventually be calculated from reported measurements (Spiegal et al., 2022). These efforts also incorporated mapping of measurement names to standardized naming conventions contained in other databases being hosted by PDI, such as the now deprecated Agricultural Collaborative Research Outcome System (AgCROS) (Delgado et al., 2018), where some historical data from sites are contained. Measurements were also mapped to the NAL thesaurus, which was designed, developed, and implemented for tagging data and metadata for ease of discovery and downloading (https://lod.nal.usda.gov/en/, accessed 8/6/2024). Version 3 of the LTAR data inventory was extended to include a new field, called LTAR_Feature_ID, which serves as a key link to standardized geospatial data, described below. Providing a key that enabled this linkage was prioritized for metrics collected in the LTAR common experiment and needed for deriving sustainable indicators of agricultural intensification.

### 2.2 Creating a controlled vocabulary for soil measurements and their attributes

Cataloging and harmonizing soil variables across all LTAR sites was the starting point for data management at the network level. Initiated in 2021 and based on Version 2 of the LTAR data inventory, a group of six scientists and a data manager established the soil data harmonization initiative. Measurements related to soils data comprise approximately 30% of the 8000 measurements listed within the LTAR data inventory. From the LTAR data inventory, a subset of soils‐related measurements was maintained on a Google Sheet worksheet for each site, which provided a working space for each site to standardize their local measurements. Guidelines were established for describing measurement names and when to converge terms that described the same measurement. A soils‐controlled vocabulary was a product of this convergence. Soil scientists were asked whether the measurement name would capture the general soil characteristic (e.g., “Extractable_Carbon”) or if additional details such as depth, methodology, and units were necessary. A second guideline was established for capturing pertinent contextual details, hereafter referred to as “soil attributes.” To avoid inadvertently recreating an existing standard of contextual attributes, scientists consulted with published references within the domain, such as the terms defined by the Soil Science Society of America (2008). Each of the 125 measurement names from the controlled vocabulary can be linked to attributes required to better understand the measurements and ensure valid cross‐site comparisons. For example, measurement of “Extractable_C” includes the filter size with the possible answer being “none,” “0.45 um,” or “other.” A Python (Python Software Foundation, 2021) script was further employed to map soil attributes to each of the site‐specific Google Sheets as actively populated dropdown menus, which users could draw upon to further document measurement attributes. The Python script dynamically updated the Google Sheets as each site mapped the soil measurements (listed in the LTAR data inventory) to the controlled vocabulary. Information from each site was then aggregated into the soil attribute discovery tool (early 2023), imported into Airtable, and embedded in the LTAR website as a dashboard.

### 2.3 Standardizing geospatial data and information

**FIGURE 3:**
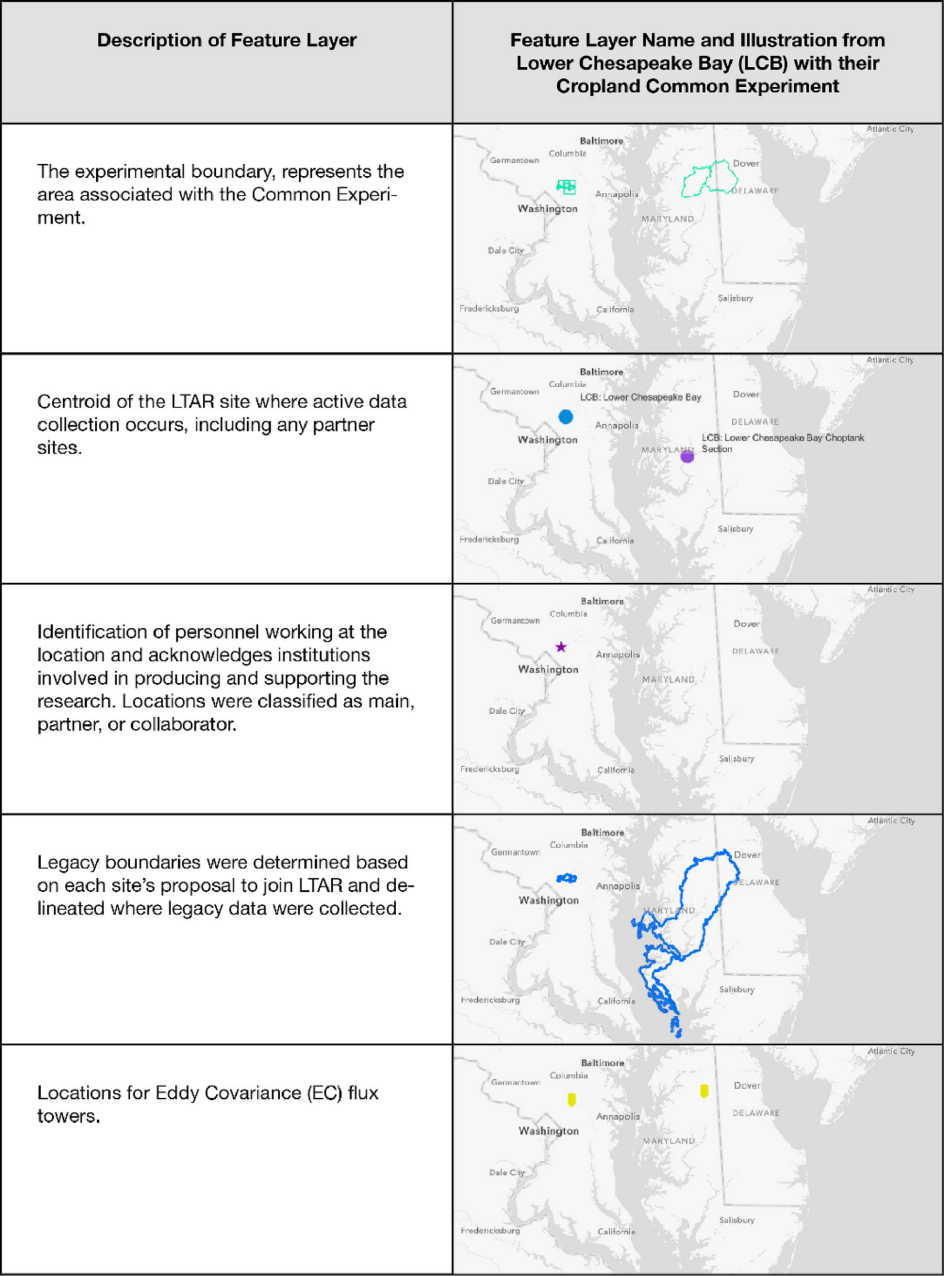
Example from the Lower Chesapeake Bay Long‐Term Agroecosystem Research (LTAR) site of the six feature layers in the LTAR standard geospatial data layers.

For the LTAR Network, implementing cross‐site, regional, and national‐level analyses requires geospatial data. To meet this need, the LTAR Remote Sensing and Geographic Information System Working Group, in collaboration with the LTAR Data Management Working Group, created the LTAR standard geospatial data layers, a set of vector data structures including polygons and points that convey rudimentary information about LTAR sites. This resulted in a set of published standardized geospatial layers representing the geographical locations of LTAR sites (Armendariz et al., 2021). A geospatial schema document was created to describe each data layer and detail the attributes of fields in the data layers; it served as a reference and a guide for the standardization process. The development team sent data requests to LTAR site leads and data managers, which were followed up with meetings to ensure the datasets conform to the common geospatial schema. The standard geodatabase serves as a single and authoritative source for key LTAR Network geospatial information. It contains six feature layers (Figure 3) (Armendariz et al., 2021), four of which are point features representing LTAR site locations, the administrative LTAR site locations, LTAR eddy flux tower locations, and LTAR camera locations participating in the PhenoCam Network. The remaining two data layers are polygon features describing the (a) historical boundaries of the LTAR site (provided when the site joined the Network) and (b) “experimental boundaries” comprising the numerous plot and field polygons where LTAR experiments are occurring across the continental United States. This latter dataset included a placeholder field for the LTAR_Feature_ID. This unique identifier would be added later to increase utility as a linkage between the standard geospatial data layers and Version 3 of the LTAR Network data inventory. All feature layers were published as a dataset in ADC (Armendariz et al., 2021) and made available on the USDA's ArcGIS Online organizational account as operational layers. Additionally, a standalone public dashboard was created in ArcGIS Online for exploration and sharing with colleagues outside the network.

### 2.4 Publishing protocols

In support of the Common Experiment, >50 LTAR researchers collaboratively standardized protocols for collection of biophysical measurements spanning plant, soil, water, greenhouse gas, biodiversity, and pest pressure. Use of standardized protocols ensures that accumulated network data are robust with the ability to compare across sites and develop systems‐level indicators. Indicators that are dependent on production and environmental data (such as yield and water quality, respectively) provide the overall motivation for collecting biophysical measurements. Scientists determined the value of each potential measurement to the LTAR Network by developing a classification schema that assigned importance as primary, secondary, or tertiary (Abendroth et al., 2024a). The criteria for a metric to be classified as primary was based on the scientific value and ability to document change within‐season and over time. The collection of primary metrics is now requested across all sites, unless significant barriers exist or they are not applicable. Protocols describing how to collect the primary metrics were subsequently written and subjected to seven steps of peer‐review and editing before publication on protocols.io (Abendroth et al., 2024a). Each protocol was assigned a DOI and then grouped together with a collection‐level DOI to facilitate discovery (Abendroth et al., 2024b). The protocols have served as the structural mechanism for data entry template design and organization. Templates are constructed for tabular time series in comma‐delimited formats with provisions for definitions of units, description of methods, and quality control mechanisms for each primary metric collected.

### 2.5 Inventorying and analyzing publications

Publication metrics and output provide a means for evaluating network productivity and influence within the scientific community over time. Six disparate lists of LTAR Network publications were identified for combination and deduplication into a single output resource: (1) a 2017 NAL Drupal website which incorporated VIVO records (Durham, 2014) and manually‐accessed library resources, with bibliographies submitted by the sites in their applications to join the LTAR Network; (2) a historical NAL bibliography, maintained using Zotero, and a second (closely aligned) list curated and maintained by NAL staff on the library website; (3) a Google Sheet maintained by LTAR data managers, which overlapped with (1) and (2); (4) a current, ongoing Zotero collection curated by NAL that incorporated automated search results for permutations of “LTAR” across multiple publication databases; (5) a list of publications on the LTAR website, which was compiled from posts by LTAR authors on the message board in Basecamp, a project management and communication platform used by the network; and (6) lists of publications, datasets, and gray literature submitted by LTAR sites and working groups via ESRI Survey123, and stored in ArcGIS Online, for LTAR annual reporting, starting in 2020.

These six lists were combined and deduplicated using Microsoft Excel and Python. The DOI was used as the unique record, or key, for each publication. Hundreds publications missing DOIs in the original lists were manually searched, and DOIs were populated for as many publications as possible. The final list of DOIs was used to query Dimensions AI, a scholarly impact tool provided by NAL, to retrieve additional details on each publication. Dimensions AI provided 41 attributes, including citation details, abstracts, acknowledgments, and measurements such as citation count, as well as two alternative metrics, relative citation ratio and field citation ratio. These are ratios of the citation count to the average citation count for the field of research and are provided for publications older than 2 years. The cleaned list of publications was then imported into Airtable and joined with the attributes from Dimensions AI.

There was a publication data gap for 2017–2020, at which time the LTAR Network sites would submit their individual bibliographies until LTAR annual reporting was initiated and an appropriate acknowledgement statement was agreed upon. This gap was filled manually by searching for the USDA's Agricultural Research Information System, for LTAR mode codes (i.e., unique identifiers for each ARS research unit). Publications from the two non‐ARS sites in the network (Archbold Biological Station, University of Florida, and the Kellogg Biological Station, Michigan State University) were collected through publicly available bibliographies on the corresponding institutional websites. NAL staff and non‐ARS site data managers manually trimmed bibliographies to contain only LTAR‐related research.

It was important that publications could be attributed to LTAR sites and discoverable by keywords related to scientific domains of expertise. A list of all LTAR participating members was acquired from Basecamp, and a Python script was applied to each publication, assigning site IDs automatically using the associated author. After several iterations of running the script and updating authors, every publication was assigned to one site or a collection of sites. Automated flags and Airtable filters helped to identify records that needed further quality control, and  publications were individually inspected to verify assigned site IDs. Publications were excluded from the final dataset if author participation in the Network could not be confirmed. The last data preparation step was to create a list of keywords for each publication. KeyBERT, which leverages the Bidirectional Encoder Representations from Transformers (BERT) deep‐learning model, was used to extract five keywords for each publication, from a combination of the title and abstract.

## 3 RESULTS

Small active teams, within the LTAR Network, directed consensus efforts on data definitions, collection methodologies, standardized metadata format and organization, and strategies for sharing data and searchable literature across research sites. Efforts to design and build these tools were essential for locating, harmonizing, mapping, describing, and sharing data and metadata across the LTAR Network. Table 1 provides a short description and links to the various tools. Teams consisted of data managers and scientists, as well as experts from NAL. At each LTAR site, a dedicated data manager is responsible for both managing site data and contributing to LTAR Network data capacity. Embedding data managers in domain‐specific working groups created opportunities to work collectively (Baker & Mayernik, 2020) and iteratively to enhance data management functionality. A community of practice was cultivated to support various research needs, as well as report progress and outcomes to program funders, network partners, and stakeholders, via online tools and data visualizations.

**TABLE 1:** Names, descriptions, and URLs of Long‐Term Agroecosystem Research (LTAR) Network tools designed and constructed to support network capacity for finding, accessing, sharing, describing, and using data.

Name of tool	Description of tool	URL
Data Inventory	A list of data produced by each site with some additional helpful information	https://ltar.ars.usda.gov/data/data‐inventory/
Soils Controlled Vocabulary and Attribute Discovery	A subset inventory of soils measurements including a controlled vocabulary for those measurements	https://airtable.com/appsb2Tl92hnqjjSL/shrBUmoc3aLA2b4Rx/tblrfVUqxVbbYDDfo/viwd4RcFbjodKYiMu
Standard Geospatial Data Layers	ESRI geodatabases and shapefiles that describe spatial features at each site	https://ltar‐usdaars.hub.arcgis.com/search?tags=sgdl
Publications Dashboard for Analysis and Metrics	An interactive list of LTAR‐related publications	https://ltar.ars.usda.gov/research/publications/
USDA ARS Long‐Term Agroecosystem Research (LTAR) Common Experiment Protocols.io	35 Protocols published by the Common Experiment	https://dx.doi.org/10.17504/protocols.io.14egn9m5ql5d/v1

Abbreviation: ESRI, Environmental Systems Research Institute.

**FIGURE 4:**
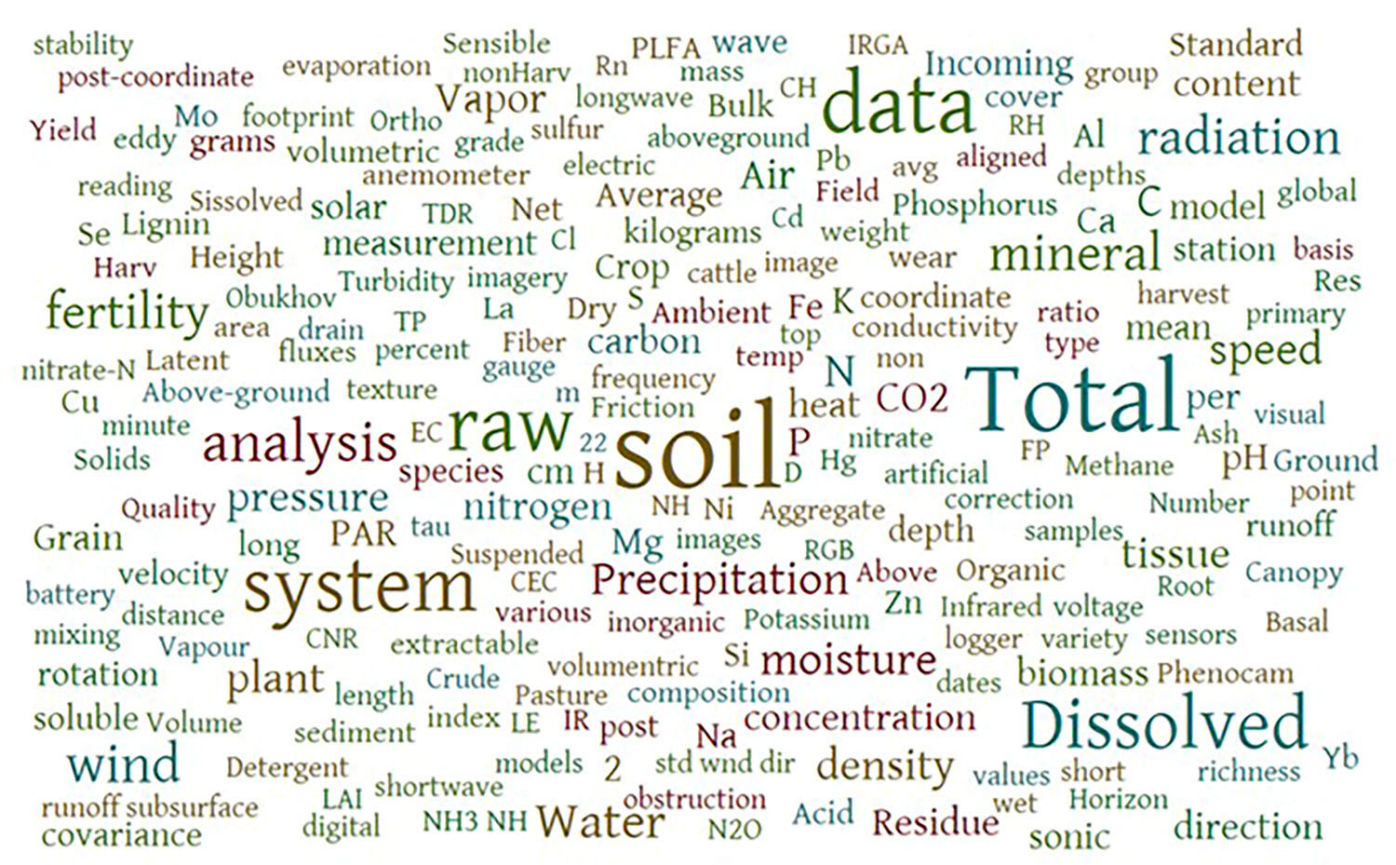
Word cloud of measurement names provided by Long‐Term Agroecosystem Research (LTAR) sites in Version 1 of the LTAR data inventory. The words pictured were used at least four times.

**FIGURE 5:**
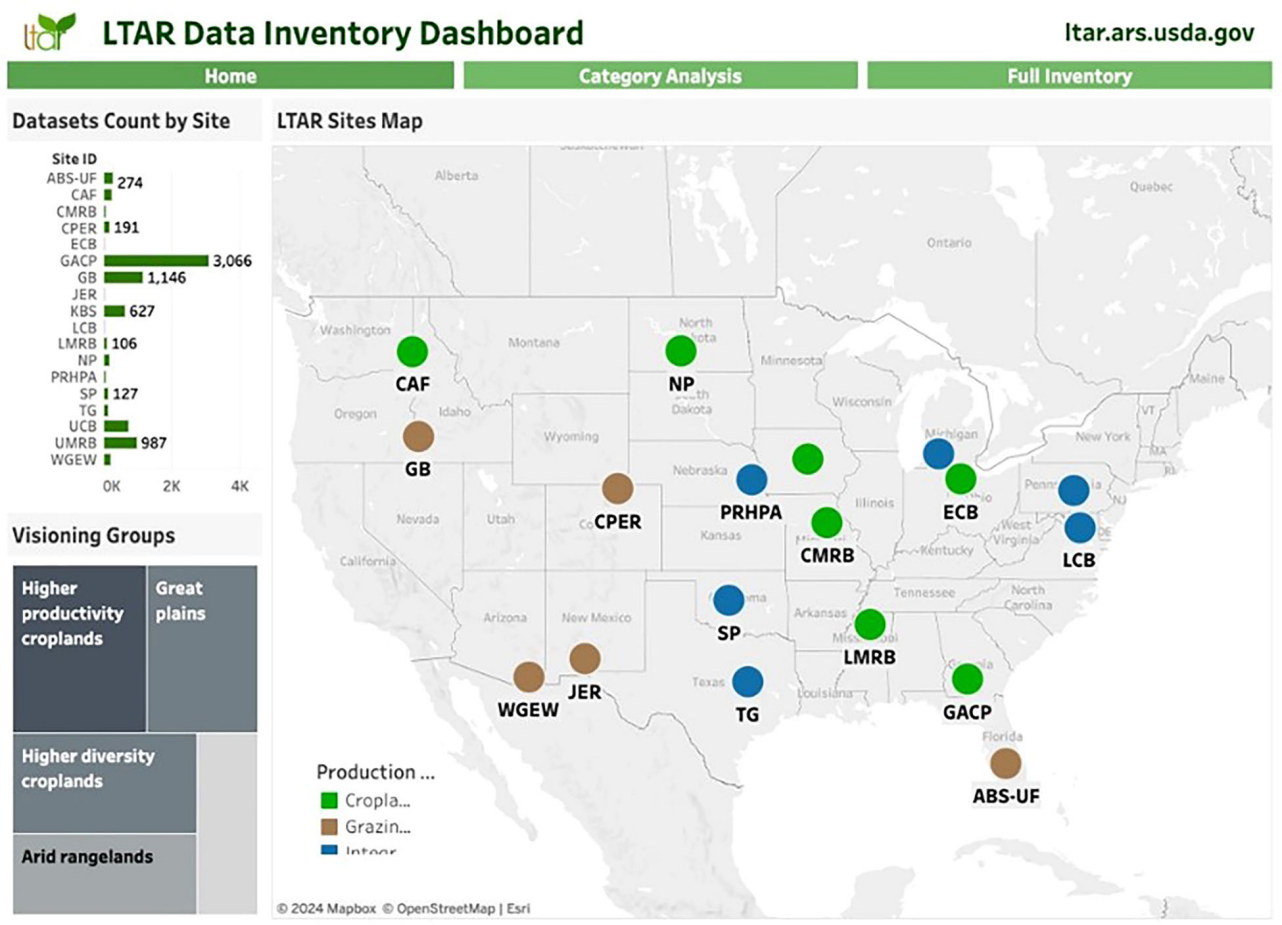
Screenshot of Long‐Term Agroecosystem Research (LTAR) data inventory dashboard in Tableau.

The first version of the LTAR data inventory allowed sites to describe their measurements using local terminology, resulting in over 6000 heterogeneous designations for 8000 reported measurement types (Figure 4). Two organizational approaches emerged from Version 1: (1) measurement consolidation into a single inventory row despite varied collection locations or projects, and (2) measurement separation by location or project. While Version 1 of the data inventory showcased multiple aggregation approaches used by the research sites, Version 2 organizational approaches produced cleaner and clearer visualizations of data collected by sites and a distinct set of categories (Figure 5) upon which to create controlled vocabularies. The controlled vocabulary for soils served as a learning opportunity and addressed the most extensive category of data, including 2584 datasets, or approximately 30% of all reported datasets in the data inventory. After several iterations, the LTAR soils working group distilled 806 terms to a controlled vocabulary of 125 terms. The terms were categorized into 26 broader themes and assigned 60 attributes essential to knowing more about each soil measurement.

Consensus building was developed through the soils controlled‐vocabulary effort, as well as the minimum requirements for cross‐site and network science metadata. Google Sheets provided a collaboration application (Karasti et al., 2010) wherein members worked on a single dataset, reverted mistakes, and commented on individual cells. However, other functionalities were still necessary for coordination of network projects, including one‐to‐many joins, publicly shareable filtered views of datasets, increased row limits, row‐specific metadata, and real‐time visualizations. Subsequently, Airtable was employed as a platform for the soils controlled vocabulary, the data inventory, and to create and manage standardized metadata related to the Common Experiment. There are 35 protocols in protocols.io, which facilitates generation of comparable data within the Common Experiment and serves as an authoritative collection of methodologies employed by LTAR researchers. Publication of the Common Experiment protocols (Abendroth et al., 2024b; Augustine & Boughton, 2024), produced an open‐access community resource to various methodologies (including protocols), standard operating procedures, and other types of practical materials and multiple format techniques to promote FAIR data.

LTAR standard geospatial data layers were used to create an authoritative geodatabase for the LTAR Network, published to USDA's ArcGIS Online enterprise account. Additionally, it was assigned a citation and DOI and made publicly available via ADC. The publication of this geodatabase satisfied new requirements outlined in the GeoSpatial Data Act of 2018 (FGDC, 2018) and the “Nelson Memo” of the USDA Office of Science and Technology Policy (OSTP, 2022), mandating the “unleashing [of] data for the greater good,” and making federally funded research data more broadly available, respectively. Publication also aligns with the USDA Geospatial Strategic Plan (OCIO, 2021), which ensures that geospatial feature layers are accurate and current, as well as openly accessible. The LTAR standard geospatial data layers enable the creation of consistent maps for use in manuscripts (Kumar et al., 2023), reports, fact sheets, webpages, and other communications.

**FIGURE 6:**
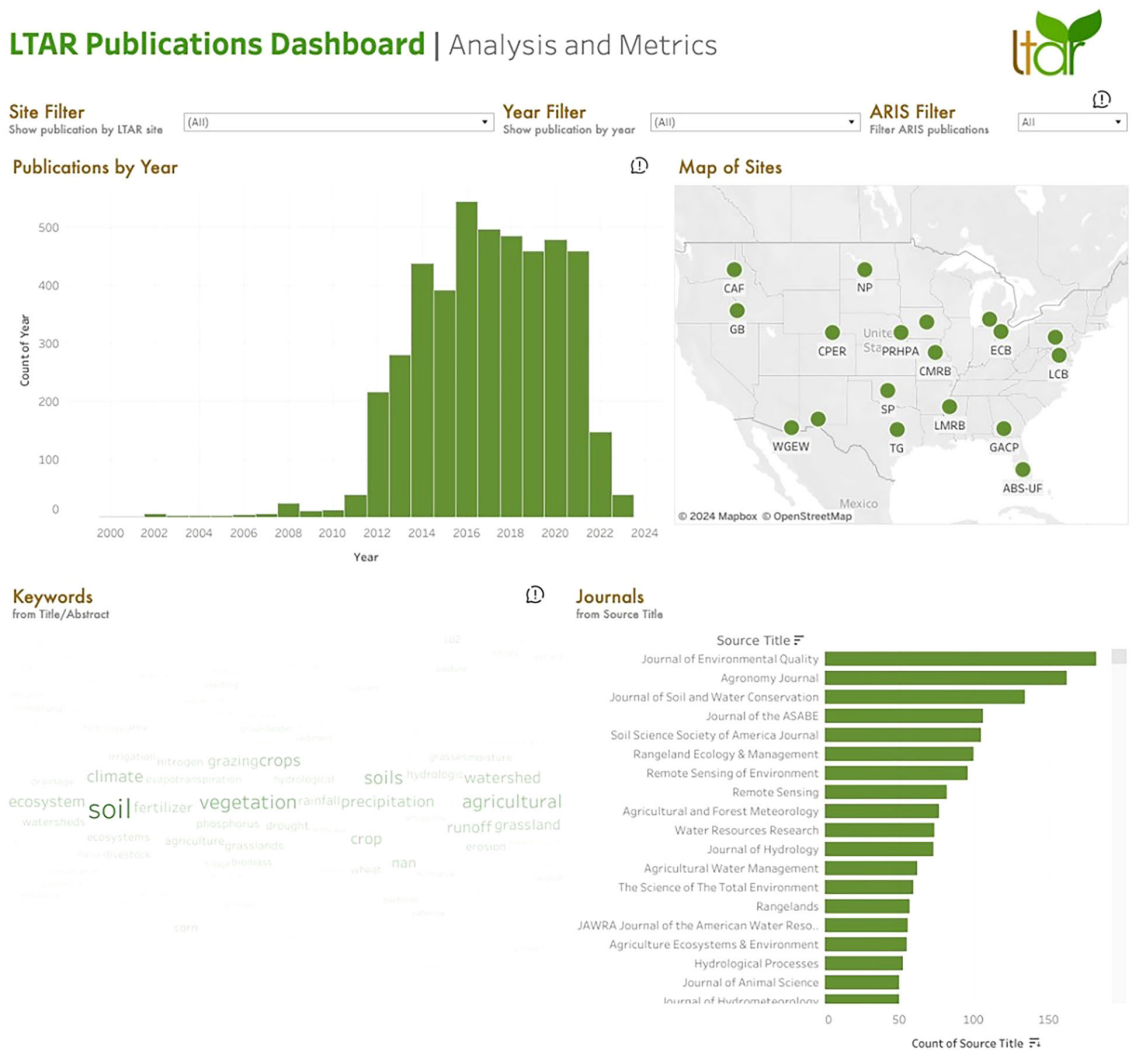
Screenshot of Long‐Term Agroecosystem Research (LTAR) publications dashboard for analysis and metrics. Bar graph summarizes publication by year as updated through 2023 and allows users to subset publications by site using filters or a dynamic map.

Development of the publications dashboard database for network output analysis and metrics leveraged the Airtable platform functionality. Publications were associated with sites, permitted track changes (i.e., manuscript additions and detail edits), and programmatically created topical categories. Tableau and Tableau Prep were used to create dynamic visualizations, such as word cloud generations, summaries of site‐specific article publications, and a ranking of the most highly cited LTAR publications. Dropdown lists in the dashboard can further narrow searches by individual site, year, author, or keyword. For each publication listed, users can click to view details and directly access the full text via a DOI link (Figure 6). It was essential for the network to disseminate information about LTAR‐related publications for assessment and visualization of its research impact. This dashboard provides access to site articles, diagrams, cross‐site and network‐wide co‐authorship, and illustrates citation metrics for its users. By using both historical and current LTAR Network publications, this comprehensive tool captures and elucidates the rich legacy of research at each site, as well as providing insights into domain expertise and collaborative efforts nationwide.

Development of subsequent versions of each of the essential tools described above included greater emphasis on linkages that would increase capacity for managing data and metadata at the network level. Tools were designed with well‐described and structured schemas, keeping in mind they would be revised and built upon to meet future network data management goals. The data inventory, soil controlled vocabulary and attribute discovery tool, and publications dashboard for analysis and metrics are now housed in an LTAR Network workspace on Airtable, which all site data managers have access to and are expected to keep current. All standardized protocols related to the LTAR Common Experiment are now published and curated within the USDA ARS portal on the protocols.io platform, which provides DOIs and thus can be cited and linked as necessary. In summary, these tools have resulted in a robust foundation of standard schemas and context upon which to build an enterprise data management system and open data portal to satisfy expectations for LTAR Network data and research.

## 4 DISCUSSION

Conducting research at a network level presents new challenges and requirements for describing, documenting, and sharing data and associated metadata for others to use. Unique and various approaches have been utilized by other programs working with data collected at multiple spatial scales and timeframes with different methodologies (Abendroth et al., 2017; Herzmann et al., 2014; Palmer, 1999). For the LTAR Network, it is not plausible for all historical data to be transformed into a standard data model. However, it is necessary for there to be useful tools within an information ecosystem (Nardi & O'Day, 2000) and that these tools are made available to a broader community interested in using historical as well as current data. This effort required collective work and consensus‐building to effectively design and create standards. Engaging in a collective process to build foundational network data management tools helped cultivate a scientific community ready and willing to share data and information.

These efforts led to metadata standards across the LTAR Network for a data inventory, common vocabulary, protocols, geospatial layers, and publication metrics while promoting solutions to network data management challenges. From a technical standpoint, standardized structure and linkages between various types of data and metadata helped to increase the efficiency of working with and visualizing data and metadata. Although some manual and time‐consuming data clean‐up was required, data standardization, aggregation, and integration were primarily performed using R and/or Python scripts due to their transparency, expandability, and reproducibility. Ultimately, this work to generate standards successfully described and harmonized measurements, and locations, and facilitated data *interoperability* and *re‐use*, addressing both *I* and *R* of the FAIR data standards. These tools enhanced the automation of metadata extraction, which enabled frequent updates to network dashboards serving the public.

As mapping applications and spatiotemporal predictive models based on big data become more ubiquitous (Peters et al., 2018, 2020), visualization of geospatial metadata and other large dynamic models are in higher demand. Dashboards provide a comprehensive visual presentation of both tabular and geospatial data and can function to enhance *findability* and *accessibility* (i.e., the *F* and *A*) of FAIR‐compliant data and information. Data visualizations also revealed significant gaps, as observed in development of the publications dashboard. Early identification of obstacles is critical for maintaining project management and completion timelines and adjusting program objectives as necessary. Dashboarding applications such as Tableau or ESRI ArcGIS Dashboards do not require software coding and provide ready‐to‐use data visualization for creating maps, legends, tables, serial charts, pie charts, indicators, and lists. Dynamic options within these dashboards (e.g., layer visibility, bookmarks, and interactive elements such as dropdown menus and filtered views) further increase their functionality for end users.

A critical gap in metadata collection and organization within the network was identified as scientists used the data inventory dashboard to find agricultural management data. These management data reference both novel strategies being tested within the LTAR Common Experiment framework, as well as prevailing practices carried out in the larger working landscapes within which these research experiments are situated. At present, there are no established tools or standard practices for describing, documenting, and sharing agricultural management practices available for the agricultural research community (Moore et al., 2022). Sites use a variety of approaches from paper files, stored at the research farm or ranch, to mobile data collection applications, being tested by PDI. Much of this information must be obtained by traditional means (i.e., contacting the site directly), as was the case for Browning et al. (2021) when studying influences of agricultural management practices timing occurring where Eddy Covariance gas fluxes were being measured and Sonnier et al. (2024) when analyzing plant communities at several LTAR sites.

Experiments that involve the manipulation of management practices will require more tools to capture the rich context associated with performing research studies and subsequently communicating about them. Development of such tools may rely on interdisciplinary team design, development, and enactment of standards. Established standards for categorizing data with controlled vocabularies can now be used to prepare data for publication in ADC, where data are grouped under LTAR and tagged with agreed‐upon terms (https://agdatacommons.nal.usda.gov/Long_Term_Agroecosystem_Research/groups). These efforts have facilitated the publication of an exemplary FAIR LTAR Network‐wide dataset (Hajek et al., 2025). Members of the network have also learned how to communicate and work collectively across fields of expertise, positioning them to address the need for documenting agricultural management practices as essential information. Much can be extrapolated and communicated about research efforts by utilizing well‐organized and documented data and metadata, and in turn, new efforts can be facilitated that rely on building upon such network data management foundations.

## 5 CONCLUSION

LTAR has made significant progress in developing foundational tools for managing and sharing data and metadata across the network. A key lesson learned was that embedding data managers in supportive roles facilitated substantial collective learning about the needs of data users. The alignment of data management practices at individual sites with network‐level standard protocols and metadata enhances data availability to the scientific community and resultant publications that answer high‐priority, customer‐relevant questions. Standardizing linkages and workflows between metadata, data, and research products increased the accessibility to, and efficiency of, working with LTAR data. This work fostered a scientific community more willing to share information, as well as establishing protocols for collecting, annotating, and disseminating that information. Additional outcomes, created with and for partners, include dynamic data exploration tools, increased online communications about the network to a broader audience, reports for administrators and stakeholders, and content for infographics. These outcomes present new avenues for discovering new collaborators or attracting funding opportunities. The foundational aspects of the LTAR data inventory, soils controlled vocabulary and attribute discovery, standard geospatial data layers, standardized protocols, and publications dashboard for analysis and metrics enhance future development of LTAR Network information management and amplify synergistic data science solutions.

## AUTHOR CONTRIBUTIONS

**Nicole E. Kaplan**: Conceptualization; data curation; methodology; writing—original draft. **Gerardo Armendariz**: Conceptualization; data curation; methodology; writing—review and editing. **Shefali Azad**: Conceptualization; data curation; methodology; writing—review and editing. **Bryan R. Carlson**: Conceptualization; data curation; methodology; writing—review and editing. **William A. White**: Conceptualization; data curation; methodology; writing—review and editing. **Lori J. Abendroth**: Writing—review and editing. **Alisa W. Coffin**: Conceptualization; data curation; methodology; writing—review and editing. **Vanessa S. Gordon**: Writing—review and editing. **Jude E. Maul**: Writing—review and editing. **William Osterholz**: Writing—review and editing. **Jonathan Sears**: Writing—review and editing.

## CONFLICT OF INTEREST STATEMENT

The authors declare no conflicts of interest.

## References

[jeq270027-bib-0001] Abendroth, L. , Herzmann, D. , Lokhande, S. , Chighladze, G. , Schwaegler, K. , & Morton, L. W. (2017). Project and research management: Integrating systems, data, and people in multidisciplinary work (Pub. No. CSCAP‐0198‐2017). Technical Report Series: Observations and Recommendations of the USDA‐NIFA funded Climate and Corn‐based Cropping Systems Coordinated Agricultural Project Vol 5 of 5. Iowa State University. https://store.extension.iastate.edu/product/15140

[jeq270027-bib-0002] Abendroth, L. J. , Liebig, M. A. , & Robertson, G. P. (2024a). USDA LTAR cropland common experiment: Standardized primary metric protocols . protocols.io 10.17504/protocols.io.261ge59pyg47/v1

[jeq270027-bib-0003] Abendroth, L. J. , Liebig, M. A. , & Robertson, G. P. (2024b). USDA ARS long‐term agroecosystem research (LTAR) common experiment . protocols.io 10.17504/protocols.io.14egn9m5ql5d/v1

[jeq270027-bib-0004] Armendariz, G. , Coffin, A. W. , Archer, D. , Arthur, D. , Bean, A. , Browning, D. , Carlson, B. , Clark, P. , Flynn, C. , Goslee, S. , Hall, V. , Holifield, C. , Hsieh, H. , Johnson, J. M. F. , Kaplan, N. , Kautz, M. , Kettler, T. , King, K. , Moglen, G. , … Yasarer, L. (2021). The long‐term agroecosystem research (LTAR) Network Standard GIS Data Layers, 2020 version . Ag Data Commons. 10.15482/USDA.ADC/1521161

[jeq270027-bib-0005] Augustine, D. J. , & Boughton, E. (2024). Aboveground net herbaceous plant production in grazinglands . protocols.io 10.17504/protocols.io.q26g71qj1gwz/v1

[jeq270027-bib-0006] Awre, C. , Baxter, J. , Clifford, B. , Colclough, J. , Cox, A. , Dods, N. , Drummond, P. , Fox, Y. , Gill, M. , Gregory, K. , Gurney, A. , Harland, J. , Khokhar, M. , Lowe, D. , O'Beirne, R. , Proudfoot, R. , Schwamm, H. , Smith, A. , Verbaan, E. , … Zawadzki, M. (2015). Research data management as a “wicked problem”. Library Review, 64(4/5), 356–371. 10.1108/LR-04-2015-0043

[jeq270027-bib-0007] Baker, K. S. , Benson, B. J. , Henshaw, D. L. , Blodgett, D. , Porter, J. H. , & Stafford, S. G. (2000). Evolution of a multisite network information system: The LTER information management paradigm. Bioscience, 50(11), 963–978. 10.1641/0006-3568(2000)050[0963:EOAMNI]2.0.CO;2

[jeq270027-bib-0008] Baker, K. S. , & Mayernik, M. S. (2020). Disentangling knowledge production and data production. Ecosphere, 11(7), e03191. 10.1002/ecs2.3191

[jeq270027-bib-0009] Browning, D. M. , Russell, E. S. , Ponce‐Campos, G. E. , Kaplan, N. , Richardson, A. D. , Seyednasrollah, B. , Spiegal, S. , Saliendra, N. , Alfieri, J. G. , Baker, J. , Bernacchi, C. , Bestelmeyer, B. T. , Bosch, D. , Boughton, E. H. , Boughton, R. K. , Clark, P. , Flerchinger, G. , Gomez‐Casanovas, N. , Goslee, S. , … Taylor, S. D. (2021). Monitoring agroecosystem productivity and phenology at a national scale: A metric assessment framework. Ecological Indicators, 131, 108147. 10.1016/j.ecolind.2021.108147

[jeq270027-bib-0010] Buchanan, E. M. , Crain, S. E. , Cunningham, A. L. , Johnson, H. R. , Stash, H. , Papadatou‐Pastou, M. , Isager, P. M. , Carlsson, R. , & Aczel, B. (2021). Getting started creating data dictionaries: How to create a shareable data set. Advances in Methods and Practices in Psychological Science, 4(1), 2515245920928007. 10.1177/2515245920928007

[jeq270027-bib-0011] Delgado, J. A. , Vandenberg, B. , Kaplan, N. , Neer, D. , Wilson, G. , D'Adamo, R. , Carter, J. , O'Gan, L. , Grow, N. , Marquez, R. , Arthur, D. , Eve, M. , Del Grosso, S. J. , Johnson, J. M. F. , Karlen, D. L. , Durso, L. , Finley, J. , Acosta‐Martinez, V. , Knaebel, D. B. , … Derner, J. D. (2018). Agricultural collaborative research outcomes system (AgCROS): A network of networks connecting food security, the environment, and human health. Journal of Soil and Water Conservation, 73(6), 158A–164A. 10.2489/jswc.73.6.158A

[jeq270027-bib-0012] Durham, S. (2014). USDA opens VIVO research networking tool to public . https://www.ars.usda.gov/news‐events/news/research‐news/2014/usda‐opens‐vivo‐research‐networking‐tool‐to‐public/

[jeq270027-bib-0013] FGDC . (2018). Federal geographic data committee . Geospatial Data Act of 2018. https://www.fgdc.gov/gda

[jeq270027-bib-0014] Gries, C. , Hanson, P. C. , O'Brien, M. , Servilla, M. , Vanderbilt, K. , & Waide, R. (2023). The environmental data initiative: Connecting the past to the future through data reuse. Ecology and Evolution, 13(1), e9592. 10.1002/ece3.9592 36620398 PMC9817195

[jeq270027-bib-0015] Hajek, O. L. , Kaplan, N. , Azad, S. , Fay, P. A. , Khorchani, M. , Nelson, A. , Schreiner‐McGraw, A. P. , Abendroth, L. , Baffaut, C. , Baker, J. , Bestelmeyer, B. T. , Boughton, E. , Browning, D. M. , Carlson, B. , Cavigelli, M. A. , Clark, P. E. , Dell, C. , Guo, Y. , Hendrickson, J. R. , … Hoover, D. L. (2025). Dataset from: Variation in patterns of production and water‐use efficiency among agroecosystems across the LTAR network . Ag Data Commons. 10.15482/USDA.ADC/26863258.v1 40714607

[jeq270027-bib-0016] Herzmann, D. E. , Abendroth, L. J. , & Bunderson, L. D. (2014). Data management approach to multidisciplinary agricultural research and syntheses. Journal of Soil and Water Conservation, Special Issue for Climate and Agriculture, 69(6), 180A–185A. 10.2489/jswc.69.6.180A

[jeq270027-bib-0017] Kaplan, N. E. , Baker, K. S. , & Karasti, H. (2021). Long live the data! Embedded data management at a long‐term ecological research site. Ecosphere, 12(5), e03493. 10.1002/ecs2.3493

[jeq270027-bib-0018] Karasti, H. , Baker, K. S. , & Millerand, F. (2010). Infrastructure time: Long‐term matters in collaborative development. Computer Supported Cooperative Work (CSCW), 19, 377–415. 10.1007/s10606-010-9113-z

[jeq270027-bib-0019] Khanna, M. , Swinton, S. M. , & Messer, K. D. (2018). Sustaining our natural resources in the face of increasing societal demands on agriculture: Directions for future research. Applied Economic Perspectives and Policy, 40(1), 38–59. 10.1093/aepp/ppx055

[jeq270027-bib-0020] Kharel, T. P. , Ashworth, A. J. , & Owens, P. R. (2022). Linking and sharing technology: Partnerships for data innovations for management of agricultural big data. Data, 7(2), 12. 10.3390/data7020012

[jeq270027-bib-0021] Kleinman, P. J. A. , Spiegal, S. , Rigby, J. R. , Goslee, S. C. , Baker, J. M. , Bestelmeyer, B. T. , Boughton, R. K. , Bryant, R. B. , Cavigelli, M. A. , Derner, J. D. , Duncan, E. W. , Goodrich, D. C. , Huggins, D. R. , King, K. W. , Liebig, M. A. , Locke, M. A. , Mirsky, S. B. , Moglen, G. E. , Moorman, T. B. , … Walthall, C. L. (2018). Advancing the sustainability of US agriculture through long‐term research. Journal of Environmental Quality, 47(6), 1412–1425. 10.2134/jeq2018.05.0171 30512071

[jeq270027-bib-0022] Kumar, J. , Coffin, A. W. , Baffaut, C. , Ponce‐Campos, G. E. , Witthaus, L. , & Hargrove, W. W. (2023). Quantitative representativeness and constituency of the LTAR network and analysis of complementarity with existing ecological networks. Environmental Management, 72(4), 705–726. 10.1007/s00267-023-01834-9 37328644 PMC10460301

[jeq270027-bib-0023] Liebig, M. A. , Abendroth, L. J. , Robertson, G. P. , Augustine, D. , Boughton, E. H. , Bagley, G. , Busch, D. L. , Clark, P. , Coffin, A. W. , Dalzell, B. J. , Dell, C. J. , Fortuna, A. , Freidenreich, A. , Heilman, P. , Helseth, C. , Huggins, D. R. , Johnson, J. M. F. , Khorchani, M. , King, K. , … Yost, J. (2024). The LTAR common experiment: Facilitating improved agricultural sustainability through coordinated cross‐site research. Journal of Environmental Quality, 53(6), 787–801. 10.1002/jeq2.20636 39406692

[jeq270027-bib-0024] Michener, W. K. , Brunt, J. W. , Helly, J. J. , Kirchner, T. B. , & Stafford, S. G. (1997). Nongeospatial metadata for the ecological sciences. Ecological Applications, 7(1), 330–342. 10.1890/1051-0761(1997)007[0330:NMFTES]2.0.CO;2

[jeq270027-bib-0025] Millerand, F. , & Baker, K. S. (2010). Who are the users? Who are the developers? Webs of users and developers in the development process of a technical standard. Information Systems Journal, 20(2), 137–161. 10.1111/j.1365-2575.2009.00338.x

[jeq270027-bib-0026] Moore, E. K. , Kriesberg, A. , Schroeder, S. , Geil, K. , Haugen, I. , Barford, C. , Johns, E. M. , Arthur, D. , Sheffield, M. , Ritchie, S. M. , Jackson, C. , & Parr, C. (2022). Agricultural data management and sharing: Best practices and case study. Agronomy Journal, 114(5), 2624–2634. 10.1002/agj2.20639

[jeq270027-bib-0027] Nardi, B. A. , & O'Day, V. (2000). Information ecologies: Using technology with heart. Mit Press. 10.7551/mitpress/3767.001.0001

[jeq270027-bib-0028] NSB . (2005). Long‐lived digital data collections: Enabling Research and education in the 21st century (National Science Board Report NSB‐05‐04). U.S. National Science Foundation. http://www.nsf.gov/pubs/2005/nsb05040

[jeq270027-bib-0029] OCIO (Office of Communications and Information Officer) . (2021). Enterprise geospatial strategic plan . https://www.usda.gov/ocio/enterprise‐geospatial‐strategic‐plan

[jeq270027-bib-0030] OSTP (Office of Science and Technology Policy) . (2022). Memorandum for the heads of executive departments and agencies . https://www.whitehouse.gov/wp‐content/uploads/2022/08/08‐2022‐OSTP‐Public‐Access‐Memo.pdf

[jeq270027-bib-0031] Palmer, C. L. (1999). Structures and strategies of interdisciplinary science. Journal of the American society for information science, 50(3), 242–253. 10.1002/(SICI)1097-4571(1999)50:3<242::AID-ASI7>3.0.CO;2-7

[jeq270027-bib-0032] Peters, D. P. C. , Burruss, N. D. , Rodriguez, L. L. , Mcvey, D. S. , Elias, E. H. , Pelzel‐Mccluskey, A. M. , Derner, J. D. , Schrader, T. S. , Yao, J. , Pauszek, S. J. , Lombard, J. , Archer, S. R. , Bestelmeyer, B. T. , Browning, D. M. , Brungard, C. W. , Hatfield, J. L. , Hanan, N. P. , Herrick, J. E. , Okin, G. S. , … Vivoni, E. R. (2018). An integrated view of complex landscapes: A big data‐ model integration approach to transdisciplinary science. Bioscience, 68, 653–669. 10.1093/biosci/biy069

[jeq270027-bib-0033] Peters, D. P. C. , McVey, D. S. , Elias, E. H. , Pelzel‐McCluskey, A. M. , Derner, J. D. , Burruss, N. D. , Schrader, T. S. , Yao, J. , Pauszek, S. J. , Lombard, J. , & Rodriguez, L. L. (2020). Big data–model integration and AI for vector‐borne disease prediction. Ecosphere, 11(6), e03157. 10.1002/ecs2.3157

[jeq270027-bib-0034] Porter, J. H. (2019). Evaluating a thesaurus for discovery of ecological data. Ecological Informatics, 51, 151–156. 10.1016/j.ecoinf.2019.03.002

[jeq270027-bib-0036] Python Software Foundation . (2021). Python language reference (version 3.10). The Python Software Foundation. http://www.python.org

[jeq270027-bib-0037] R Core Team . (2021). R: A language and environment for statistical computing (version 3.6.0). R Foundation for Statistical Computing. https://www.R‐project.org/

[jeq270027-bib-0038] Robertson, G. P. , Allen, V. G. , Boody, G. , Boose, E. R. , Creamer, N. G. , Drinkwater, L. E. , Gosz, J. R. , Lynch, L. , Havlin, J. L. , Jackson, L. E. , Pickett, S. T. A. , Pitelka, L. , Randall, A. , Reed, S. , Seastedt, T. R. , Waide, R. B. , & Wall, D. H. (2008). Long‐term agricultural research: A research, education, and extension imperative. Bioscience, 58(7), 640–645. 10.1641/B580711

[jeq270027-bib-0039] Short, N. M. , Woodward‐Greene, M. J. , Buser, M. D. , & Roberts, D. P. (2023). Scalable knowledge management to meet global 21st century challenges in agriculture. Land, 12, 588. 10.3390/land12030588

[jeq270027-bib-0048] Soil Science Society of America . (2008). Glossary of soil science terms 2008. ASA‐CSSA‐SSSA.

[jeq270027-bib-0040] Sonnier, G. , Augustine, D. J. , Paudel, S. , Porensky, L. M. , Silveira, M. , Toledo, D. , Azad, S. , Boughton, R. K. , Browning, D. M. , Clark, P. E. , Fay, P. A. , Kaplan, N. , Thibault, K. M. , Swain, H. M. , Veum, K. S. , & Boughton, E. H. (2024). Impact of plant diversity and management intensity on magnitude and stability of productivity in North American grazing lands. Applied Vegetation Science, 27(2), e12776. 10.1111/avsc.12776

[jeq270027-bib-0041] Spiegal, S. , Webb, N. P. , Boughton, E. H. , Boughton, R. K. , Brymer, A. L. B. , Clark, P. E. , Collins, C. H. , Hoover, D. L. , Kaplan, N. , McCord, S. E. , Meredith, G. , Porensky, L. M. , Toledo, D. , Wilmer, H. , Wulfhorst, J. , & Bestelmeyer, B. T. (2022). Measuring the social and ecological performance of agricultural innovations on rangelands: Progress and plans for an indicator framework in the LTAR network. Rangelands, 44(5), 334–344. 10.1016/j.rala.2021.12.005

[jeq270027-bib-0042] Teytelman, L. , Stoliartchouk, A. , Kindler, L. , & Hurwitz, B. L. (2016). Protocols. io: Virtual communities for protocol development and discussion. PLoS Biology, 14(8), e1002538. 10.1371/journal.pbio.1002538 27547938 PMC4993360

[jeq270027-bib-0043] Thibault, K. M. , Laney, C. M. , Yule, K. M. , Franz, N. M. , & Mabee, P. M. (2023). The US National Ecological Observatory Network and the Global Biodiversity Framework: National research infrastructure with a global reach. Journal of Ecology and Environment, 47(21). 10.5141/jee.23.076

[jeq270027-bib-0044] Waide, R. B. , Brunt, J. W. , & Servilla, M. S. (2017). Demystifying the landscape of ecological data repositories in the United States. Bioscience, 67(12), 1044–1051. 10.1093/biosci/bix117

[jeq270027-bib-0045] Waller, D. M. , & Flader, S. (2010). 3 Leopold's Legacy. In I. Billick & M. V. Price (Eds.), The ecology of place: Contributions of place‐based research to ecological understanding (pp. 40–62). The University of Chicago Press.

[jeq270027-bib-0046] Wickham, H. (2014). Tidy data. Journal of Statistical Software, 59(10), 1–23. 10.18637/jss.v059.i10 26917999

[jeq270027-bib-0047] Wilkinson, M. D. , Dumontier, M. , Albersberg, I. J. , Appleton, G. , Axton, M. , Baak, A. , Blomberg, N. , Boiten, J. W. , da Silva Santos, L. B. , Bourne, P. E. , Bouwman, J. , Brookes, A. J. , Clark, T. , Crosas, M. , Dillo, I. , Dumon, O. , Edmunds, S. , Evelo, C. T. , Finkers, R. , … Mons, B. (2016). The FAIR guiding principles for scientific data management and stewardship. Scientific Data, 3(1), Article 160018. 10.1038/sdata.2016.18 26978244 PMC4792175

